# Insights into the pan-microbiome: skin microbial communities of Chinese individuals differ from other racial groups

**DOI:** 10.1038/srep11845

**Published:** 2015-07-16

**Authors:** Marcus H. Y. Leung, David Wilkins, Patrick K. H. Lee

**Affiliations:** 1 School of Energy and Environment, City University of Hong Kong, Hong Kong

## Abstract

Many studies have characterized microbiomes of western individuals. However, studies involving non-westerners are scarce. This study characterizes the skin microbiomes of Chinese individuals. Skin-associated genera, including *Propionibacterium*, *Corynebacterium*, *Staphylococcus*, and *Enhydrobacter* were prevalent. Extensive inter-individual microbiome variations were detected, with core genera present in all individuals constituting a minority of genera detected. Species-level analyses presented dominance of potential opportunistic pathogens in respective genera. Host properties including age, gender, and household were associated with variations in community structure. For all sampled sites, skin microbiomes within an individual is more similar than that of different co-habiting individuals, which is in turn more similar than individuals living in different households. Network analyses highlighted general and skin-site specific relationships between genera. Comparison of microbiomes from different population groups revealed race-based clustering explained by community membership (Global R = 0.968) and structure (Global R = 0.589), contributing to enlargement of the skin pan-microbiome. This study provides the foundation for subsequent in-depth characterization and microbial interactive analyses on the skin and other parts of the human body in different racial groups, and an appreciation that the human skin pan-microbiome can be much larger than that of a single population.

The skin is the first line of defense against injury from the unpredictable external environment, and an intricate nature of the skin is the presence of a myriad of microorganisms including bacteria, fungi, viruses, and mites, reaching concentrations of over 10^7^ cells/cm^2^ 1. This conglomerate of life forms, most of which are of commensal nature, constitutes the skin microbiome responsible for preventing colonization and invasion by pathogens and modulation of innate and adaptive immunities. Subsequently, various medical and allergic conditions are associated with perturbations and alterations in one’s skin microbial community^2^,^3^,^4^,^5^.

The skin itself is a dynamic environment, as different physical, chemical, and physiological properties vary across skin sites and individuals^6^. A corollary to such endogenous properties of the skin is the selection of different microbial communities according to topography of the skin ecosystem^7^. Also, skin microbiomes of a given site can be different depending on host genetic properties, as well as demographic and other personal attributes including gender, age, race, sanitary practices, lifestyles, and physical injury, and changes to microbial communities may occur as rapid as within hours and minutes^8^,^9^,^10^,^11^,^12^, underscoring the dynamics of the skin microbiome.

With the advent of cultivation-independent sequencing technology, appreciations for skin microbiome investigations across individuals and skin sites have increased^7^,^8^,^13^,^14^. The Human Microbiome Project (HMP) has extensive collection of data, generating some of the most informative reports of skin microbiome using high-throughput sequencing^15^. However, most of these studies involve western subjects, and reports analyzing the effect of race in skin microbiome is limited to a handful of studies^9^,^15^,^16^. Given that racial and geographical differences may affect skin properties, it should come as no surprise that skin property variations will select for different sets of microbial communities, as seen in other parts of the human body^17^,^18^. In particular, sequence-based skin microbiome studies of individuals from Asia, especially China, where close to 20% of the world’s population live, is limited to a single study with limited demographic breadth and sequencing depth^19^. We believe that a skin microbiome investigation of Chinese individuals on a greater scale and across different demographic properties will increase our limited understanding on the relationship between the skin microbial life forms and the health and well beings of this prominent but overlooked population. Also, an increased understanding of skin baseline microbiomes across different population groups should lead to comparative analyses between microbiomes and health and diseases across different groups, ultimately providing clinical interventions taking demographic factors into account. Furthermore, should individuals from different racial population groups vary in microbiomes, the skin microbiome across racial boundaries should give rise to a “pan-microbiome” much larger than previously thought and appreciated.

In this work, two hundred-skin samples from Chinese individuals living in Hong Kong were analyzed. We demonstrate that the bacterial diversity and community composition present extensive individual diversity that can partially be explained by various demographic factors. Specifically, this work identifies genera that may act as major drivers of community differences between population groups, including *Enhydrobacter*, which we postulate to be especially enriched in Chinese individuals and potentially interacting with other microorganisms. More importantly, despite the similar taxonomic and community diversity trends observed compared to other studies, microbial membership and structure comparison between our cohort and other works reveals strong and significant clustering patterns based on race.

## Results

### Taxonomic overview of Chinese skin microbiomes

**Figure 1:**
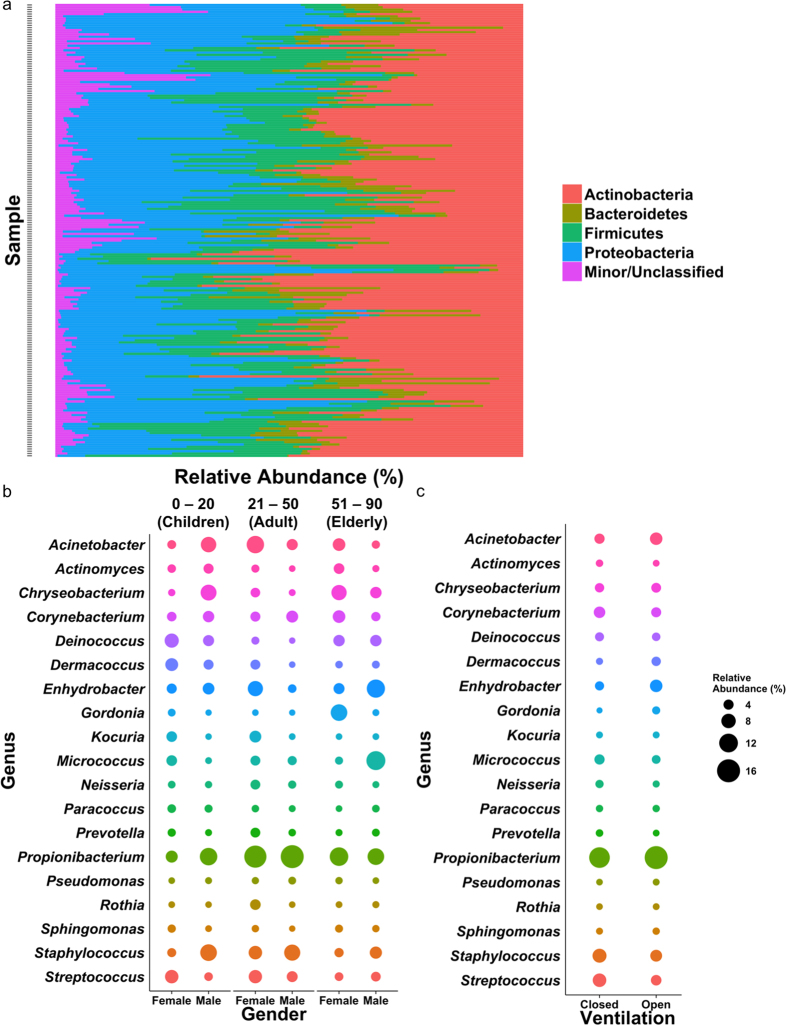
Taxonomic breakdown of skin microbiomes of Chinese descents in Hong Kong. (**a**) Relative abundances of the top four phyla across 200 samples included in this study, with samples arranged alphabetically. (**b**,**c**) Bubble plots of the relative abundance of the top genera within samples grouped by (**b**) gender and age groups, and (**c**) ventilation mode of household. Relative abundance represented as sizes of bubbles as indicated in legend. Abundance data and statistics can be found in Supplementary Data 2.

Among 200 skin samples (Supplementary Data 1), a total of 7,192,068 reads passing quality control were clustered into 62,606 distinct OTUs based on 97% sequence identity cutoff (following dataset singleton removal), the majority (82.2%) of which were *de novo* with <97% sequence identity with known OTUs. The dataset contained 52 phyla, 137 classes, and 750 genera. The top four phyla, *Actinobacteria* (36.6%), *Proteobacteria* (31.6%), *Firmicutes* (19.1%), and *Bacteroidetes* (7.1%) made up over 94% of all reads (Fig. 1a). At the genus level, the top 10 genera on average make up >53% of the microbial community within each sample, and well-documented skin colonizers *Propionibacterium*, *Staphylococcus, Acinetobacter, Streptococcus, Enhydrobacter* and *Corynebacterium* were detected on all individuals. However, their relative abundances differed by up to more than 1,000 folds within a site across individuals (Supplementary Table 1), with the palm regions exhibiting the widest relative abundance range. The abundances of some genera also differed by gender; males generally showed higher abundances of *Propionibacterium*, *Staphylococcus*, *Enhydrobacter*, whereas *Streptococcus* was more abundant in the female population (Fig. 1b and Supplementary Data 2 for abundance and statistical data). Genus-level comparison was also performed for members of different age groups, revealing significant age-driven average abundance variations between some of the abundant genera. Skin-associated genera were also more abundant in occupants of households without natural ventilation during time of sampling, and individuals residing in naturally ventilated households contained higher abundances of *Enhydrobacter* and *Gordonia* (Fig. 1c, Supplementary Data 2). Although *Propionibacterium* was on average more abundant in individuals exposed to open ventilation, this was not significant (Mann-Whitney (MW) *p* = 0.11).

### Non-core genera constitutes majority of skin microbial communities

Of the 750 genera detected in total, core genera (defined as those that were detected in all household/individual) represented a minority of the microbial composition (178/750, 23.7% for household data and 104/750, 13.9% for individual data); the majority of the genera were detected in some but not all households/individuals (distributed genera), and those that are found in only one household/individual (unique genera) represent more than 10% of the entire community, indicating the extensive community heterogeneity across samples, individuals, and households (Supplementary Data 3). The most diverse skin site, the left forearm, also contained the highest number of unique OTUs (Supplementary Data 3). Extensive site-diversity was also observed; a total of 28,471 (45%) OTUs were detected on only one of the five skin sites (Supplementary Data 3).

### Presence of potential opportunistic pathogens within *Staphylococcus* and *Streptococcus*

Some OTUs detected here belong to genera containing opportunistic pathogens. In-depth taxonomic understanding is therefore crucial in unraveling species-level diversity of these genera, so as to ascertain both the presence as well as the proportions of potential pathogens present on the skin of our population group. *Staphylococcus* and *Streptococcus* were selected for analysis because they constitute species of clinical importance, and are the focus of other microbiome investigations where species-level identification is relevant^15^,^20^.

Proportions of *Staphylococcus* and *Streptococcus* ranged from 0.1% to >40% and 0.2% to >28%, respectively, and significantly differed between households (Kruskal-Wallis (KW) *p* = 2.2 × 10^−16^ for both genera). We selected sequences classified as the two genera (total of 864,066 reads containing 471,450 and 392,616 staphylococcal and streptococcal reads, respectively), and interrogated against the PathoSystems Resource Integration Center (PATRIC) database for species-level identification. A total of 459,399 (97.44%) staphylococcal and 355,554 (90.56%) streptococcal reads were retained and classified as species. These reads were assigned into 17 staphylococcal and 47 streptococcal species (Supplementary Data 4).

**Figure 2:**
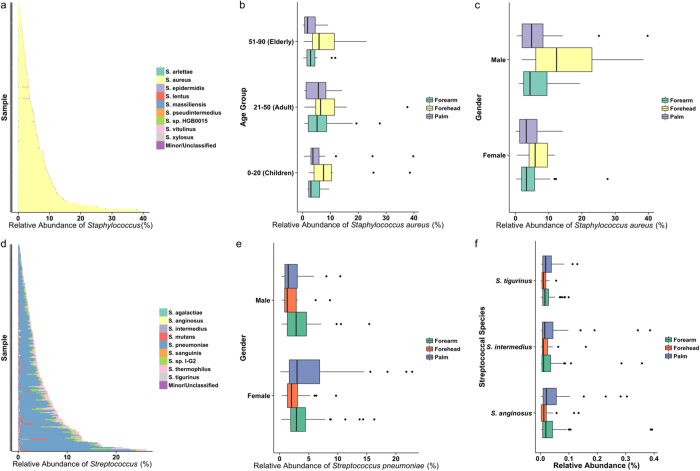
Relative abundances of *Staphylococcus* and *Streptococcus* species. (**a**) Relative abundance of (**a**) staphylococcal and (**b**) streptococcal species plotted against each sample, ordered by increasing abundance. Each bar represents the abundance of genus as a percentage of the entire microbial community, with different colours within a bar representing one of the top nine individual species/strains, with remaining species within each genus grouped as “Minor/Unclassified.” Box-and-whisker plots (split by skin sites) of *S. aureus* expressed as a percentage of the entire microbial community across (**c**) age groups, and (**d**) genders. (**e**) Box-and-whisker plots of *S. pneumomiae* relative abundance expressed as a percentage of the entire microbial community across gender, separated by anatomical sites. (**f**) Box-and-whisker plots of relative abundances of minor but potentially pathogenic streptococcal species by skin site, expressed as a percentage of the entire microbial community. All classified staphylococcal and streptococcal (respectively) species are listed in Supplementary Data 4.

Sequences resembling the potential human pathogen *S. aureus* overwhelmingly dominated the staphylococcal population across samples (Fig. 2a), with only a handful of skin samples containing smaller proportions of *S. xylosus* and *S. arlettae*, the latter being more abundant on foreheads and dominant palms of some individuals. One common skin colonizer and potential pathogen, *S. epidermidis*, contributed to an average of <1 % of the staphylococcal population. Significant differences in *S. aureus* abundance was observed between age groups (KW *p* = 0.006), but post-hoc pairwise comparisons between the groups revealed that this was due to significantly lower abundance of this species in the elderly population compared to the adult group (average *S. aureus* abundance of 4.2% in the elderly compared to 6.8% and 6.7% in the children and adult groups, respectively). Forehead samples also had higher abundances of *S. aureus* compared to forearm and palm sites (when left and right samples were combined) (KW *p* = 0.0003, post-hoc comparisons showed significant differences when comparing forehead sites) (Fig. 2b). The opportunistic pathogen was also significantly more abundant in males (MW *p* = 0.007) (Fig. 2c).

The *Streptococcus* genus presented more species-level diversity (Fig. 2d), but appeared to be lower than that of the oral cavities^15^. *Streptococcus pneumoniae* (the pneumococcus) overall presented the majority of identified species of this genus, ranging from a proportion of 30%–89% of the total streptococcal population within a given sample. The pneumococcus was significantly more abundant in females (average abundance of 4.2% in females compared to 3.0% in males, MW *p* = 0.02), but did not significantly differ by anatomy (KW *p* = 0.12) (Fig. 2e). The predominance of the pneumococcus on skin is different from other human sites, where related species such as *S. mitis* cluster, including members of *S. pneumoniae*, *S. mitis*, *S. oralis*, and *S. pseudopneumoniae*, predominate in the nares^11^,^21^ and the more distant relative *S. parasanguinis* on the tongue^15^. *Streptococcus mutans*, a causative agent of dental caries^22^, was present in considerable proportions in some sites in this study (this species alone represents up to 4% of the entire microbial communities in some samples). A yet unidentified species with strain name *S.* sp. I-G2^23^ was also among the most abundant streptococcal species on skin. Interestingly, *Streptococcus pyogenes*, a common causative agent of skin infection^24^, was present in <1% of the streptococcal population. The emerging streptococcal respiratory and invasive pathogen *Streptococcus tigurinus* was present in low (average of 0.02% of total microbiome across all samples belongs to *S. tigurinus*) proportions (Fig. 2f)^25^.

### Factors associated with sample (α-) diversity differences

**Figure 3:**
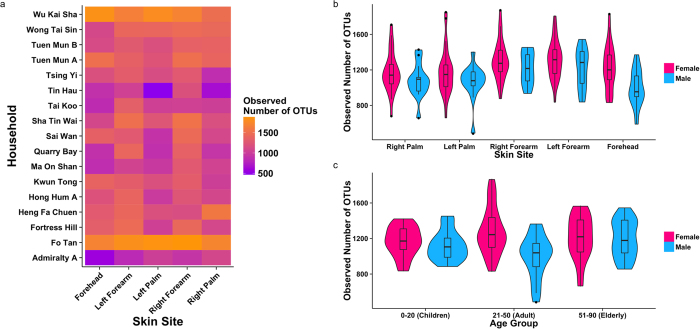
α-diversity analyses based on observed number of OTUs. (**a**) Heat map of observed rarefied number of OTUs for household (indicated by location) and skin site. Violin plots and super-imposed box-and-whisker plots showing median and quartile ranges of observed rarefied number of OTUs between male and female samples grouped by (**b**) skin site and (**c**) age group. All α-diversity values were calculated based on rarefaction of 13,240 reads per sample to account for differences in sequencing depth between samples.

Taxonomy-based (observed number of OTUs and Chao1 estimator) and phylogeny-based (Faith’s Phylogenetic Diversity, FPD) indices were employed to assess factors associated with differences in α-diversity. The lack of plateaus in rarefaction plots based on observed OTUs may suggest under-sampling (Supplementary Fig. 1), however Good’s coverage estimations of over 92% across samples suggest sufficient sampling depth to capture the majority of the microbial diversity (Supplementary Data 5). After normalization, α-diversity was significantly different between households, skin sites (regardless of whether symmetrical sites were combined for analysis), gender, and household ventilation across all three α-diversity indices (Fig. 3 and Supplementary Table 2). Based on MW pairwise and KW multiple and post-hoc pairwise comparisons tests, forearms, when combining both left and right sites, were significantly more diverse than both palm and forehead areas, but differences between symmetrical forearm and palm sites were insignificant. In addition, females presented greater diversity compared to men across sites (greatest gender differences at the foreheads) and age groups. α-diversity was only significant between different age groups using Chao1 estimator, suggesting that α-diversity differences between age groups is driven by sample singleton OTUs.

### Specific genera drive differentiation of β-diversity between population and sample groups

β-diversity was computed based on weighted (assessment of community structure by considering abundance of OTUs) and unweighted (assessment of community membership by considering only OTU presence/absence) UniFrac distances, and revealed that household, age group, ventilation, and skin sites were significant clustering predictors for both community structure and membership (Table 1). While household location, age, and skin site has been shown to affect skin microbiomes^11^,^15^,^26^, the role of household ventilation on occupant skin microbiome is less clear, and the relationship between indoor residence microbiomes and that of occupant skin may be more complex^27^. Differences between households can be more explained by community membership, in that groupings of communities between families can be more explained by the mere presence and absence of OTUs, given that the unweighted Global R value for household is almost double that of weighted Global R value. When considering samples by age groups, the clustering magnitude is modestly greater when considering weighted UniFrac distances, consistent with previous works suggesting that age-related changes in microbiomes on skin over time is predominantly based on changes in microbial members already established at earlier ages^11^,^28^. When symmetrical left and right skin sites were combined, significance was not seen using unweighted analysis, suggesting that differences in microbial communities between left and right sites are mainly derived from differences in abundances of OTUs mostly present on both sides, rather than distinct presence/absence of particular OTUs on one of the two symmetrical sides for a given site. Other factors, such as smoking habits, handedness, and presence of pets, did not explain community variations on occupant skin, however this could be due to the insufficient statistical power in comparing these variables due to the low number of samples within each category.

**Figure 4:**
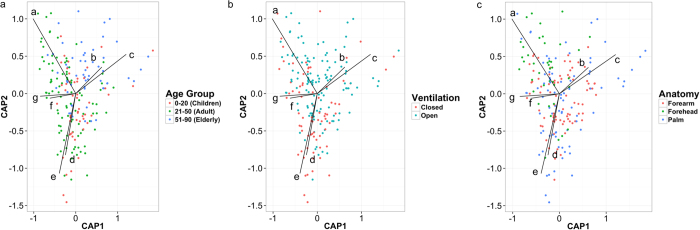
Weighted UniFrac distance-based redundancy analysis (DB-RDA) constrained by relative abundance of major genera. PCoA plots generated based on grouping of samples by (**a**) age group, (**b**) household ventilation mode, and (**c**) anatomical site. UniFrac DB-RDA for the three plots are constrained by common genera as follows: a* = Propionibacterium*, b = *Enhydrobacter*, c = *Chryseobacterium*, d = *Acinetobacter*, e = *Neisseria*, f = *Streptococcus*, g = *Corynebacterium*, h = *Staphylococcus*. CAP1 and CAP2 represent, respectively, the first and second constrained axes used in the CAP (canonical analysis of principal coordinates).

Distance-based redundancy analysis (DB-RDA) shows that potential major genera driving community differentiation (Fig. 4) include *Propionibacterium*, *Staphylococcus*, *Streptococcus*, *Corynebacterium*, and *Neisseria* seen across the adult population, and *Enhydrobacter* and *Chryseobacterium* within elderly individuals. Also, *Propionibacterium* OTUs also drives the clustering of forehead communities, and OTUs belonging to *Enhydrobacter* and *Chryseobacterium* drove skin microbial community clustering of individuals living in households with open natural ventilation. The relatively high abundance of this genus on the forehead is consistent with a previous study^7^, most likely because of the preference of lipophilic members of this genus residing on sebaceous surfaces.

### Effects of co-habitation on similarities in skin microbial community structures

To test whether individuals living together present more similar microbial community structures as seen in previous works^26^,^28^, weighted UniFrac distances were compared between individuals living within the same and in different households. The mean intra-individual weighted UniFrac distance (0.177) was lower than that of different individuals within the same household (mean distance = 0.246), which was smaller than individuals living in different households (mean distance = 0.296) (KW *p* = 2.2e^−16^, post-hoc test reveals significance between all pairwise comparisons) (Table 2). When analyzed by different skin sites, mean UniFrac distances were also greater for comparisons between individuals of different households across all sites, with the greatest differences in palm sites regardless of symmetry. This is not surprising, as palms are likely to be the sites where the individuals interact mostly with their immediate environments by touching, in addition to variations in palm physiologies^8^, and cohabiting individuals are likely to come into contact with the identical surfaces within household communities.

Density plots (Supplementary Fig. 2) by skin site (combining symmetrical sites) show bimodal distributions for comparisons within individuals when combining symmetrical forearm sites. The reason for this distribution is not entirely clear, and does not appear to be governed by the number of individuals co-habiting in the household of the particular individual. Double peaks were not observed for comparison within households when left and right sites were analyzed separately (Supplementary Fig. 3), and between different individuals, whether they were living together or not, suggesting that personal variations in microbial structures (regardless of whether they are living together or not) were significant enough to conceal any symmetry-based differences. Consistent to this, microbial communities on the same side between different individuals were not more similar than when comparing sites of opposing symmetry for both forearms (MW *p* = 0.07) and palms (MW *p* = 0.09). However, communities between symmetrical sites within the same individuals were still more similar than that of between individuals within households and between households (Table 2).

### Network analyses reveal general and site-specific co-abundance between genera

**Figure 5:**
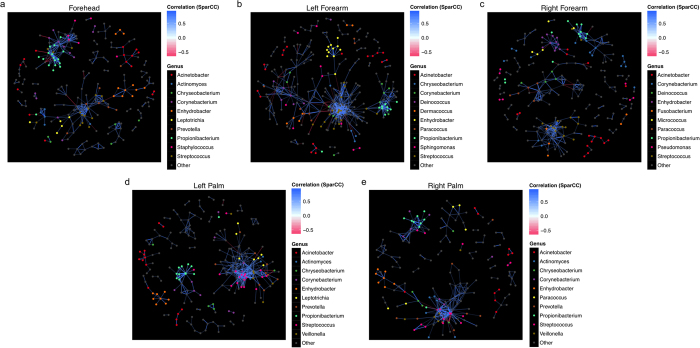
SparCC network plots of co-abundance and co-exclusion correlations between OTUs by skin sites. Separate network plots were constructed for (**a**) forehead, (**b**) left forearm, (**c**) right forearm, (**d**) left palm, and (**e**) right palm. Nodes represent OTUs involved in either significant co-abundance (blue edges) or co-exclusion (red edges) relationships, with the magnitude of the correlation expressed as the intensity of the respective edge colours. The colour of each node indicates the genus of the OTU. Only significant correlations (two-sided pseudo *p* ≤ 0.05 based on bootstrapping of 100 repetitions) with an absolute correlation magnitude ≥0.6 are presented both for visual clarity and to allow focus of only strong correlations, given only intra-site correlations are considered. The top 10 genera most involved in significant correlations are listed, and the remaining genera are grouped as “Other.” All significant relationships (both co-abundance and co-exclusion) provided in Supplementary Data 6.

SparCC was employed to highlight co-abundance relationships between OTUs for each skin site^29^. Over 400,000 significant relationships were observed in total (Supplementary Data 6), with the co-abundance to co-exclusion ratios close to one for all sites, similar to findings of Faust *et al.*^30^ when considering within-site relationships. Also, forearm sites with higher α-diversities also showed a greater number of significant interactions. Co-abundance network for each site, with strong and significant (i.e. SparCC correlation magnitude of ≥0.6, *p* ≤ 0.05) nodes and edges between the top eight genera for each site plotted (Fig. 5). Comparison of the sites reveals interesting inter-genus correlations that are either site-independent or site-specific. Specifically, some site-independent connections include the overall positive interactions between OTUs of *Propionibacterium* and *Corynebacterium* and co-exclusive clusters containing OTUs of *Enhydrobacter* and *Streptococcus*, as well as a lack of apparent significantly strong interaction of *Acinetobacter* OTUs to other genera. However, these same members interact with different genera depending on skin location. This is exemplified in the significantly strong co-abundance relationships between *Enhydrobacter* and *Acinetobacter* OTUs on left palms, and the exclusion of *Enhydrobacter* and *Micrococcus* on right forearms. In addition, a large number of nodes throughout the networks are OTUs of “minor” genera (grey) that are relatively lower in abundance.

Positive correlations were modestly more common between genetically related OTUs of the same genus, while the opposite is true for OTUs between genera (Supplementary Fig. 4). The mean SparCC values for inter-genus relationships for forehead, left forearm, right forearm, left palm, and right palm were all below zero (i.e. −0.0035, −0.0029, −0.0033, −0.0034 and −0.0031, respectively), and were significantly different from that of the mean intra-genus SparCC values, which are all positive (0.10, 0.10, 0.11, 0.14, and 0.13 for forehead, left forearm, right forearm, left palm, and right palm respectively, MW tests for corresponding sites were *p* < 2.2 x 10^−16^ for all sites). However, genus-by-genus and site-by-site examinations reveal that co-abundance involving members of *Enhydrobacter* on foreheads were especially dominated by intra-genus relationships (average SparCC magnitudes of 0.35 and −0.017 for intra- and inter-genus interactions, respectively, MW *p* < 2.2 x 10^−16^) (Supplementary Fig. 5). In contrast, the mean intra- and inter-genus SparCC magnitudes for *Corynebacterium* are more similar to that of the general relationships when all genera are considered (0.051 and −0.0012 for intra- and inter-genus, respectively, MW *p* < 2.2 × 10^−16^).

### Microbial community differences between racial groups reveal a larger “pan-microbiome”

**Figure 6:**
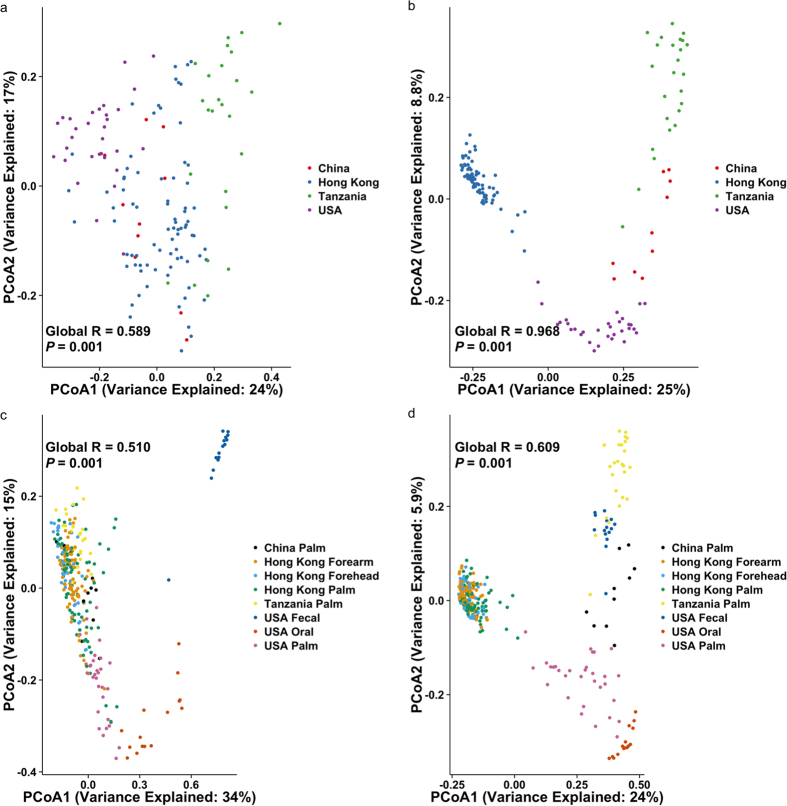
Principal coordinated analysis of skin microbiomes from different studies. Human microbiome data from China^19^, USA^13^, and Tanzania^9^ were included in the analysis. PCoA plots based on (**a**) weighted and (**b**) unweighted UniFrac distances of palm communities as well as other (**c**,**d**) skin, oral and fecal communities indicate both community structure (weighted, a and c) and membership (unweighted, b and d) variations. Respective ANOSIM Global R values showing the extent of community variation between the compared sample groups and statistical significance are indicated. Axes represent the two dimensions explaining the greatest proportion of variances in the communities for each analysis.

A limited number of investigations characterizes skin microbiomes from non-western individuals^9^,^16^,^19^. In this study, publicly available data of samples from China^19^, USA^13^ and Tanzania^9^ were included and compared with our Hong Kong cohort using weighted and unweighted UniFrac analysis. As all these other studies only involved palm samples, separate analyses and principal coordinated analysis (PCoA) plots were performed involving palm samples (Fig. 6a,b). Both abundance-weighted (Global R = 0.589, *p* = 0.001, Fig. 6a) and unweighted (Global R = 0.968, *p* = 0.001, Fig. 6b) analyses reveal strong clustering associated with geographical and/or racial differences even only considering palm samples. Interestingly, microbiomes from the two Chinese studies, including subjects leading different lifestyles (undergraduates living in dormitories in subjects enrolled in the work of Ling *et al.*^19^ compared to individuals across a wide age range and lifestyles in this study) conducted in different laboratories and with different sequencing platforms clustered together in the weighted analysis.

When combined with other microbiomes (including fecal and oral samples from the study of Caporaso *et al.*^13^ and our forearm and forehead samples), weighted UniFrac PCoA analysis revealed that skin microbiomes of the different cohorts were more clustered than microbiomes of other host sites (Fig. 6c). When comparing communities based solely on membership (i.e. unweighted UniFrac analysis, Fig. 6d), racial differences in microbial community membership within a skin site could be greater than that of different biogeographical sites within a racial group (for example, microbiomes of palms and oral cavities of Americans may be more similar than between palm samples between Americans, Hong Kong Chinese, and Tanzanians) (Fig. 6d). Overall, these observations suggest that both the presence/absence and the abundances of the OTUs explain any potential geographical/racial differences on skin microbiomes, and that the absence/presence of OTUs found on the same skin site across geography/races may not necessarily be less different than that of different skin sites.

**Figure 7:**
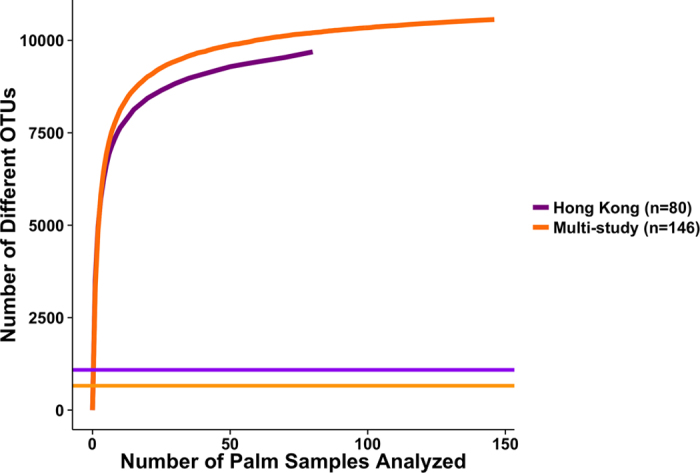
Number of distinct OTUs plotted against number of additional left and right palm samples analyzed. Palm samples from within Hong Kong only (n = 80, purple curve) as well as additional samples from Hong Kong, China, USA, and Tanzania (n = 146 in total, orange curve) included for analysis. Horizontal line represents average number of OTUs per sample present for Hong Kong sample group (1,087, purple line) and multi-study group (658, orange line).

To evaluate how addition of different population groups contributes to enlarging the skin “pan-microbiome”, the number of distinct OTUs present in each additional inclusion of a palm sample (regardless of symmetry) is plotted (Fig. 7). Compared to the averages of 1,087 and 658 OTUs per sample (horizontal lines) for the Hong Kong-only samples and multi-study samples (including samples from Hong Kong, China, USA, and Tanzania) respectively, the repertoire of OTUs increased as the analyzed sample number increased, regardless whether only Hong Kong palm samples (orange curve) or all four studies (purple curve) were included. Specifically, compared to only considering Hong Kong palm samples, inclusion of samples from other studies increased the number of OTUs detected. The lack of plateauing for both curves suggests yet additional OTUs can be discovered upon analyzing more samples. Nonetheless, we show that, even within a single site, the pan-microbiome of individuals across population and racial groups is magnitudes greater than any single sample.

## Discussion

This is the first large-scale skin microbiome investigation dedicated to Chinese people, which make up nearly 20% of the world’s population, and also the first ever to systematically compare the skin microbiome of Asian with that of other populations. It must be noted, however, that the current cohort selected constitute simply one part of the Chinese population. It is likely that, other population groups within China, with their wide variety of environmental exposure, diet, and lifestyles, will show skin microbiome structure differences, as shown in the gut^31^,^32^. Having said that, community observations here reveal general broad observations in line with other Western studies^7^,^8^,^11^,^13^,^15^. Specifically, the predominance of a limited number of the common bacterial phyla and skin-associated genera, the inter-personal taxonomic and phylogenetic community variations, and the roles of skin sites, gender (physiology or lifestyle-based^33^), and households in taxonomic abundances and α-/β-diversities have been documented. However, we show that skin microbial communities vary between different racial backgrounds, which cannot be explained by methodological variations alone. Both community membership and structure contributed to differences in microbial communities between racial groups, ultimately enlarging the global pan-microbiome. Some of the variations can be explained by presence or absence of specific OTUs that may be low in abundance. This is exemplified in the observation that, when only analyzing presence/absence of OTUs (unweighted UniFrac analysis), community membership between sites within a population group can be more similar than that of the same site between groups. While this is counter-intuitive to previous works showing site-specific community differences^34^, unweighted analyses would magnify the effect of OTUs present in low abundances. When OTU abundances are taken into account (weighted UniFrac analysis), the site-specific clustering in overall community structure appears to be greater than that of population groups, indicating that the most abundant members of a given site is generally similar across population groups. While it is unknown whether these “minor” microbial members are transient or long-term skin colonizers from this study, these OTUs may play roles in defining the microbiomes of different cultural backgrounds, as illustrated in the strong clustering based on unweighted UniFrac. Furthermore, these OTUs may be involved in potential ecological interactions with other microbial members. Also, understanding the temporal dynamics of rarer microbial members may further enlighten their roles on skin, as they may vary in abundances over time in responses to rapid changes in their surroundings^35^.

Common opportunistic pathogens including *S. aureus* and *S. pneumoniae* were detected in this Chinese cohort. *S. aureus* is among the leading bacterial causative agents of skin infections^36^. Given a sizable fraction of *S. aureus*-mediated skin infections in the US^37^ and a recent *S. aureus* outbreak in Hong Kong^38^ due to methicillin-resistant strains, high prevalence of *S. aureus* on the skin in this study is worrisome, and although the individuals in this study are asymptomatic, this observation warrants further surveillance given the role of these organisms in skin, respiratory, and invasive infections. Similarly, one should caution the ubiquity and abundant nature of the pneumococcus on the skin of this population, as pneumococcal respiratory and invasive diseases are among the most common clinical complications following influenza infections, a situation that is notably serious in Hong Kong^39^. It must be noted, however, that using the 16S rRNA gene to classify streptococcal species is problematic, as intra-species 16S rDNA sequence variation in this genus may be greater than the OTU clustering threshold of 3% sequence variation, and related *Streptococcus* species may share up to 99% sequence identity over the entire 16S gene^40^. Nonetheless, further metagenomic analysis will be beneficial in further understanding resistance profiles and virulence potentials of these potential pathogens, as well as a greater understanding of recently characterized (*S. pseudopneumoniae* and *S. tigurinus*) and unclassified species (such as *S.* sp. I-G2) and their roles on the skin.

This study provided further support that *Enhydrobacter* are common on the skin of Chinese individuals. This genus was initially isolated from a lake^41^, and have since been detected in some built environments^42^,^43^ and in individuals with blepharitis^44^. However, one should not discredit the outdoor nature of this genus, especially when this genus is shown to drive clustering of communities from occupants of naturally ventilated households. Further investigation of its strong and significant relationships, both between members within *Enhydrobacter*, as well as with other genera (namely *Streptococcus*), on different skin sites are therefore beneficial.

SparCC network analysis suggests that not only members of the microbial communities, but also their co-abundance and co-exclusion relationships, differ between sites. Furthermore, genera possess variations in both inter- and intra-genus relationships for particular skin sites. Previous studies, either by network analysis on human skin microbiomes or *in vitro* and *in vivo* models^5^,^45^ suggest that metabolisms of *Propionibacterium* and *Staphylococcus* inhibit each other’s ability to colonize the skin. However, strong co-exclusion interactions between these two genera were not detected here. In fact, co-abundance relationship between them is detected on the forehead of this cohort. Therefore, potential interactions observed in particular studies might only be specific to the population group in question. The networks also highlight the potential importance of “minor” genera in the overall microbial interaction across the human skin. We do not have temporal data to describe whether these minor members represent transient “occupants” of the human skin or permanent members present in low abundance. What is known, based on western studies, is that microbial membership rapidly changes, and microbial population dynamics is subjected to skin sites and differences in the extent of microbial diversity within individuals^7^,^8^,^46^. Therefore, it is also difficult to predict whether and how community structure changes overtime in different population and racial groups. A recent work highlighted that the individualities of skin microbiomes over time was closely related to baseline diversity of individuals^46^. Specifically, individuals within a population with higher microbial community diversity will also experience greater microbial dynamics over temporal scales. Given that microbial diversity variations can be observed between population groups with different lifestyles^16^, comparison of microbial community dynamics between population groups living in distinct locations and leading different lifestyles will shed light into the relationships between human and their immediate environments. Having said that, it is probable that factors governing the extent of microbial changes within an individual involve wide-scope ethnic/racial (particular diets for gut microbiomes, living conditions, etc.), as well as specific personal (frequency of showering and hand washing, use of cosmetics and skin care products, etc.) attributes. Hence, clustering of the extent of microbial temporal dynamics by racial groups may not be as well defined, and differences in microbial dynamics may still be greater between individuals than between population groups.

This study demonstrated that incorporation of skin microbiomes from different racial population groups increases the microbiome by magnitudes even within a site. Much research of the past decade examines the healthy state of the skin, and recently its comparison with the microbiomes of hosts with skin conditions. However, this work suggests that the knowledge regarding skin microbiome from previous works potentially represents only a fraction of global citizens, and large-scale microbiome studies provide minimal information, if any, about racial backgrounds of the sampled subjects^33^,^46^,^47^. This has serious implications: interventions in attempts to improve one’s health by altering the microbiomes may not be effective for different population groups as a result of varying baseline microbiomes. However, while there is an association between race and microbiome differences, it is unlikely that race itself directly drives variations. Rather, race- and culture-dependent host physiologies and other factors may combine to shape microbiome changes, as both endogenous (genetics) and exogenous factors (diet and interaction with the environment) shape human microbial assemblages^9^,^16^,^18^.

In summary, this study provided evidence that the Chinese present distinct skin microbiomes from other populations, despite shared microbiome trends. The human skin microbiome thus is present as a “pan-microbiome”, larger than any microbial community of an individual or a group. While the number of studies compared here to portray the pan-microbiome size is limited, we anticipate that the size of the pan-microbiome will expand as microbiome information of additional unique population groups come into light. Also, we anticipate that appreciation for the greater pan-microbiome across different parts of the human body will lead to future investigations dedicated to populations of different racial, ethnic, and cultural backgrounds, enabling better assessments of whether current microbiome knowledge can be or should be applied to all population and racial groups.

## Materials and Methods

### Subject and household experimental design

A total of 40 individuals of Chinese decent (none of which were offspring of interracial marriages) and long-term residents of Hong Kong were involved in this study, which was a part of a larger work commenced in January 2014 analyzing the relationships between air, surface, and occupant skin microbiomes across various households in Hong Kong^27^. Ethics approval for subject sampling was granted by the City University of Hong Kong Ethics Committee (reference number 3-2-201312 (H000334)). Sampling and all subsequent steps described in the Materials and Methods have been conducted in accordance with relevant guidelines. All subjects of this study were asymptomatic during sampling, and have not had taken antibiotics three months prior to the sampling. The individuals in this study were living in 17 households throughout rural and urban parts of Hong Kong to cover a broad local geographical scope (Supplementary Data 1). Individuals and household were selected to cover a range of age and lifestyle choices such as smoking, pets, and allergies. All households involved in this study did not use pesticide or have purchased new furniture up to three months prior to sampling. After being informed about the nature of the study, as well as their roles and responsibilities as subjects, written informed consent was given by all individuals. Each household filled a questionnaire to record individual and household characteristics (Supplementary Data 1).

### Skin swab sampling

For each individual, five skin sites (forehead, left and right forearms, left and right palms) were swabbed. These sites were selected to facilitate multi-study comparison, as previous studies also analyzed the aforementioned sites^9^,^13^,^19^. In brief, autoclaved swabs were moistened with a sterile swab solution (0.15M NaCl with 0.1% Tween 20)^8^ and each surface was sampled for 15 seconds by swapping the cotton tip along the surfaces in back-and-forth motions. Samples were subsequently stored in −80 °C within one hour of sampling and until DNA extraction to minimize organism growth post-sampling^43^.

### DNA extraction, PCR of 16S rRNA gene region, library construction and sequencing

Genomic DNA (gDNA) extraction and PCR amplification (triplicate reactions for each sample) were performed as described previously^43^. Extracted gDNA was sent to Health GeneTech Corporation (Taoyuan City, Taiwan) for library preparation and sequencing on an Illumina MiSeq. Illumina adapters were attached to amplicons using the Illumina TruSeq DNA sample preparation kit v2. Purified libraries were applied for cluster generation and sequencing on the Illumina MiSeq platform to generate paired-end 150 bp reads. Blank swab controls were included and processed in the gDNA extraction, library preparation, and sequencing stages in parallel for contamination control (see below).

### Sequence and bioinformatics analyses

FASTX-Toolkit (http://hannonlab.cshl.edu/fastx_toolkit) and the QIIME pipeline (v.1.8.0)^48^ were used to process the raw sequences, and sequence chimera filtering was performed with ChimeraSlayer via the QIIME “parallel_identify_chimeric_seqs.py” script. Non-chimeric sequences with a minimum acceptable Phred quality score of 20 in terminal bases and ≥20 for 70% of their length were retained for downstream analysis. Following quality filtering and trimming, sequences shorter than 100-bp were removed. Forward reads were used for this analysis. Unless otherwise described, data and statistical analyses were performed using R and Perl scripts. High-quality sequences were clustered into operational taxonomic units (OTUs) against the Greengenes rRNA gene sequence database (ftp://greengenes.microbio.me/greengenes_release/gg_13_5/gg_13_8_otus.tar.gz; 97% rep set), using the UCLUST-based open-reference OTU clustering pipeline implemented in QIIME’s “pick_open_reference_otus.py” script, with a 97% sequence identity cutoff. Reads with <97% sequence identity were allowed to form *de novo* clusters without taxonomic classification. OTU lineages with average relative abundances of >0.5% in the blank samples were considered as contaminants and were removed from samples. As part of the QIIME pipeline, a pre-clustering filtering step is used such that data sequences below 60% identity to the reference data set are removed. Global singleton OTUs were removed to account for potential sequencing artifacts. For downstream steps requiring phylogenetic tree construction (Faith’s phylogenetic diversity [FPD] and UniFrac distances), representative sequences for OTU clusters were selected based on the most abundant sequence within each cluster and aligned against the Greengenes reference alignment using PyNAST^49^, as implemented in the QIIME script “align_seqs.py”. In addition, chloroplast, chimeric and other sequences that failed to align were removed.

To generate rarefaction curves for α-diversity analyses (observed OTUs, FPD, and Chao1 total richness estimator), ten increments of sampling depth between 10 and the sample median depth of 37,460 were selected, and the averages of ten repeated richness measurements were calculated as implemented in QIIME script “alpha_rarefaction.py”. α-diversity analyses described in the results are based on the normalized depth of 13,420 reads per sample to account for differences in sequencing depths. For β-diversity analyses, distance-based redundancy analysis (DB-RDA) based on weighted (assesses community structure) and unweighted (assess community membership) UniFrac distances was computed using the capscale function in the R package vegan (http://vegan.r-forge.r-project.org/), where the relative abundances of the major genera *Propionibacterium*, *Enhydrobacter*, *Neisseria*, *Chryseobacterium*, *Corynebacterium*, *Streptococcus*, and *Staphylococcus* were inputted as variables. β-diversity was also compared between our dataset and other works^9^,^13^,^19^ using closed-reference OTU cluster formation (based on ≥97% sequence identity to Greengenes database as described above but without *de novo* clustering, due to different 16S rRNA gene region analyzed for different studies, and different sequencing technology with different read depths). The resulting Unifrac distance matrices were plotted as principal coordinated analysis (PCoA) and both weighted and unweighted analysis of similarities (ANOSIMs) were determined using the R package vegan. For species-level analysis of potential pathogens, OTUs classified as the *Staphylococcus* and *Streptococcus* genera by UCLUST were interrogated with the Pathosystems Resource Integration Center (PATRIC) database^50^, containing curated 16S rRNA sequences and associated species-level taxonomic information. Through the QIIME USEARCH clustering algorithm, sample reads were classified into species if they share ≥97% sequence identity with PATRIC database reads.

SparCC was employed to represent co-abundance and co-exclusion networks between taxa, as it does not take into account the relative proportions of taxa in the samples (i.e. community composition of the samples) but the absolute abundance of taxa^29^. SparCC and calculation of two-sided pseudo *p* values (*p* values ≤ 0.05 considered significant) were run on python scripts based on bootstrapping of 100 repetitions. A network plot was generated for each of the five skin sites, and correlation magnitudes ≥0.6 (indicating strong co-abundant relationships) and ≤−0.6 (indicating strong co-exclusion relationships) were plotted.

### Statistical analyses

The nonparametric Mann-Whitney (MW) and Kruskal-Wallis (KW) tests were employed to determine significance when comparing between two or more comparison groups, respectively. Where indicated in the main text, post-hoc KW pairwise comparison tests for significance between individual groups were performed using the kruskalmc function in R package pgirmess (http://cran.r-project.org/web/packages/pgirmess/index.html) following significant KW observations.

### Sequence deposition

Raw reads in fastq format and metadata file for this project have been uploaded previously along with other samples included in a separate study unraveling the inter-relationships between household air, surface, and occupant skin microbiomes of Hong Kong onto FigShare (http://dx.doi.org/10.6084/m9.figshare.1254031)^27^. Samples included in this particular study are indicated as “Skin” under the “Type” column in the accompanying metadata.

## Additional Information

**How to cite this article**: Leung, M. H. Y. *et al.* Insights into the pan-microbiome: skin microbial communities of Chinese individuals differ from other racial groups. *Sci. Rep.* **5**, 11845; doi: 10.1038/srep11845 (2015).

## Supplementary Material

Supplementary Data 1

Supplementary Data 2

Supplementary Data 3

Supplementary Data 4

Supplementary Data 5

Supplementary Data 6

Supplementary Information

## Figures and Tables

**Table 1:** ANOSIM Global R values based on weighted and unweighted UniFrac distances of microbial community between variables.

Variable	Weighted Global R	*P*value^a^	Unweighted Global R	*P*value^a^
Household Location	0.363	0.001	0.665	0.001
Age Group	0.160	0.001	0.143	0.001
Ventilation	0.0768	0.003	0.0611	0.01
Skin Site (left/right separate)	0.0528	0.001	0.0150	0.02
Skin Site (combining symmetry)	0.0399	0.013	0.0122	NS
Gender	0.0350	NS	0.0366	NS
Pets	−0.0733	NS	−0.000252	NS
Smoking	−0.0157	NS	−0.0505	NS
Handedness	−0.135	NS	0.0667	NS

^a^NS: statistically non-significant (*P* > 0.05).

**Table 2:** Average within-site weighted UniFrac distances between individuals based on households (HHs).

Comparison	Same individual^a^	Individuals within HHs	Individuals between HHs	Statistic^b^	*P*value	Post-hoc test^c^
**Skin (combined)**	0.177	0.246	0.296	KW	<0.001	Highly significant across all comparisons
**Forehead**	ND	0.233	0.250	MW	0.03	None
**Forearm (combined)**^d^	0.108	0.221	0.264	KW	<0.001	Highly significant across all comparisons
**Left**	ND	0.224	0.265	MW	<0.001	None
**Right**	ND	0.217	0.263	MW	<0.001	None
**Palm (combined)**^d^	0.123	0.289	0.334	KW	<0.001	Highly significant across all comparisons
**Left**	ND	0.294	0.337	MW	<0.001	None
**Right**	ND	0.289	0.331	MW	<0.001	None

^a^ND: no data as only one site within each individual.

^b^KW: Kruskal-Wallis (comparison with more than two variables), MW: Mann-Whitney (comparison with two variables).

^c^Post-hoc multiple pairwise comparisons performed for significant KW analysis using kruskalmc function in pgirmess R package.

^d^Left and right samples for a given site are combined for pairwise UniFrac distance calculations. Distances between sites within an individual are all comparisons between left and right sites within an individual.
