# Erratum: Insights into the pan-microbiome: skin microbial communities of Chinese individuals differ from other racial groups

**DOI:** 10.1038/srep21355

**Published:** 2016-02-25

**Authors:** Marcus H. Y. Leung, David Wilkins, Patrick K. H. Lee

Scientific Reports 5: Article number: 1184510.1038/srep11845; published online: 07162015; updated: 02252016.

The original version of this Article contained errors in the legend of Figure 2, where text was not matched to the correct subpanel.

The legend now reads:

**Relative abundances of** ***Staphylococcus*** **and** ***Streptococcal*** **species**. (a) Relative abundance of *Staphylococcus* species plotted against each sample, ordered by increasing abundance. Each bar represents the abundance of genus as a percentage of the entire microbial community, with different colours within a bar representing one of the top nine individual species/strains, with remaining species/strains grouped as “Minor/Unclassified.” (b,c) Box-and-whisker plots (split by skin sites) of *S. aureus* expressed as a percentage of the entire microbial community across (b) age groups and (c) gender. (d) Relative abundance of *Streptococcus* species plotted against each sample, ordered by increasing abundance, similar to staphylococcal plot in Fig. 2a. (e,f) Box-and-whisker plots of relative abundances of minor but potentially pathogenic streptococcal species by skin site, expressed as a percentage of the entire microbial community. All classified staphylococcal and streptococcal species are listed in Supplementary Data 4.

In Supplementary Figures 4 and 5, the density plots depicting the correlation magnitude curves were incorrectly provided.

The legend of Figure 4 now reads:

**Inter- and intra-genus co-abundance and co-exclusion magnitude comparisons.** Density plots of SparCC co-abundance and co-exclusion magnitudes between OTUs of the different (red) and same (blue) genera. Magnitude >0 and <0 represents co-abundance and co-exclusion relationships respectively. Plots generated for (a) forehead, (b) left and (c) right forearm, and (d) left and (e) right palm sites.

The legend of Figure 5 now reads:

**Inter- and intra-genus significant relationships between (a)** ***Enhydrobacter*** **and (b)** ***Corynebacterium*** **on the forehead.** Density plots of SparCC significant *Enhydrobacter* and *Corynebacterium* co-abundance and co-exclusion magnitudes between OTUs of the different (red) and same (blue) genera. Magnitude >0 represents co-abundance relationships, where <0 represents co-exclusion relationships.

These errors have been corrected in the Supplementary Information that now accompanies the Article.

In addition, the data from row 65536 onwards was truncated in Supplementary Dataset 6. These errors have been corrected in the Supplementary Dataset 6 file that now accompanies the Article.

