# Supplementary material for: Insights into the pan-microbiome: skin microbial communities of Chinese individuals differ from other racial groups
Source: Sci Rep. 2015 Jul 16;5:11845. doi: 10.1038/srep11845 (PMC4503953; doi:10.1038/srep11845)
Supplement: Supplementary Information [file srep11845-s7.docx]

**Supplementary Information**

Insights into the pan-microbiome: skin microbial communities of Chinese individuals differ from other racial groups

Marcus H. Y. Leung, David Wilkins, Patrick K. H. Lee

**Supplementary Figures**


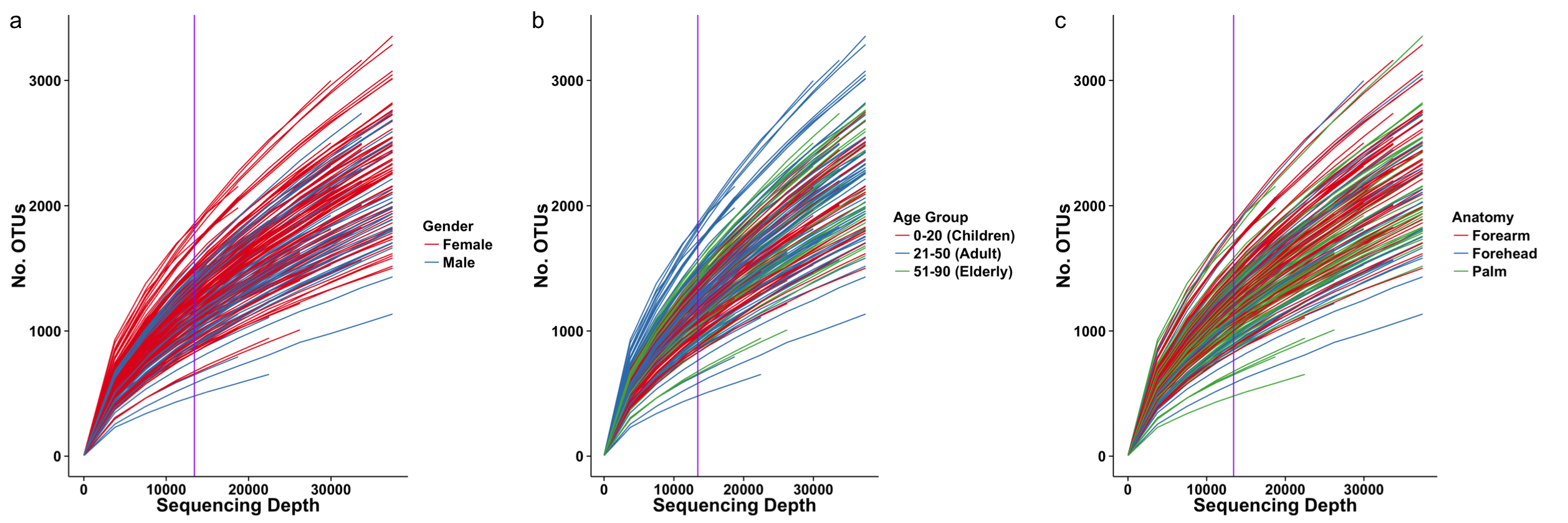


**Supplementary Fig. 1:** **Rarefaction plots on observed number of OTUs.** Number of OTUs tabulated based on ≥ 97% cut-off to Greenegenes database is plotted against number of sequence reads (sequencing depths) for each sample. Samples are coloured by (a) gender, (b) age group, and (c) sampling site. For all plots, the maximum depth shown in figure is 37,460 reads per sample, corresponding to the median read depth across all samples before rarefaction. Ten increments between depths of 10 and 37,460 reads inclusive were selected, and for each increment, the average richness of ten measurements was used to construct each curve. α-diversity comparisons performed in main text were based on a normalized read depth of 13,420 reads per sample (purple vertical line) to account for read depth differences between samples.


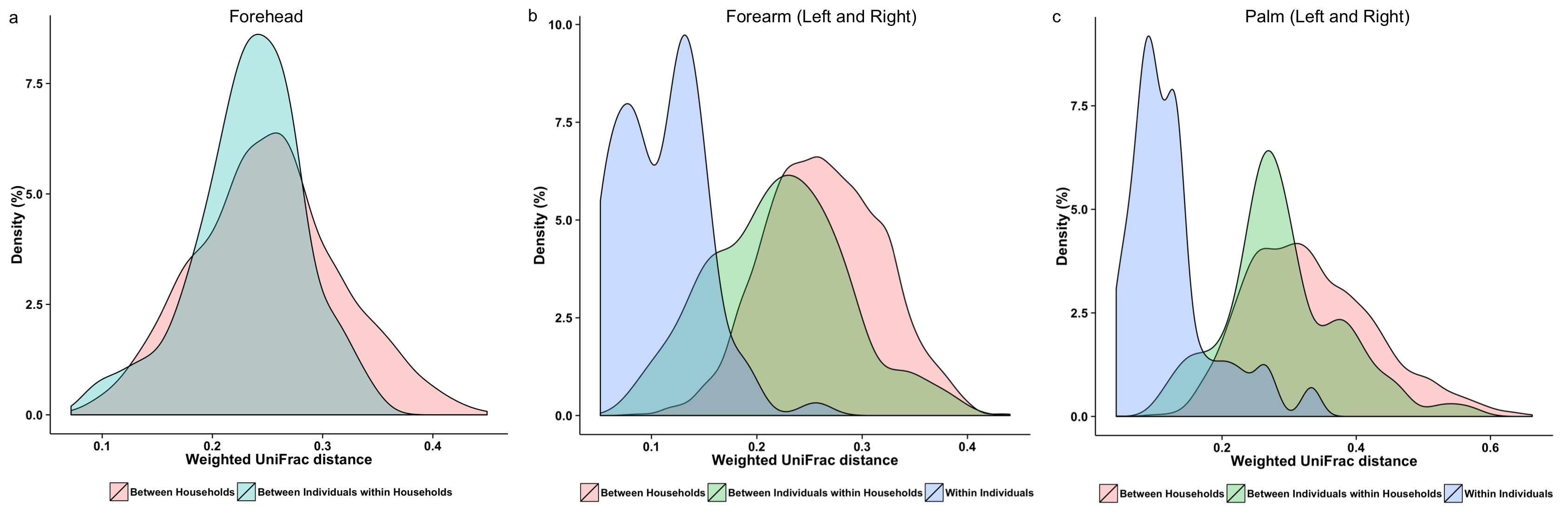


**Supplementary Fig. 2: Community structure dissimilarity between individuals by symmetry-combined skin sites.** Density plots of community structure dissimilarities represented by weighted UniFrac phylogenetic distances between individuals for (a) forehead, (b) symmetry-combined forearm, and (c) symmetry-combined palm sites. Areas under curves are colour shaded depending on the relationships of samples under comparison (i.e. samples from same individuals, samples between individuals within a household, or samples between households).


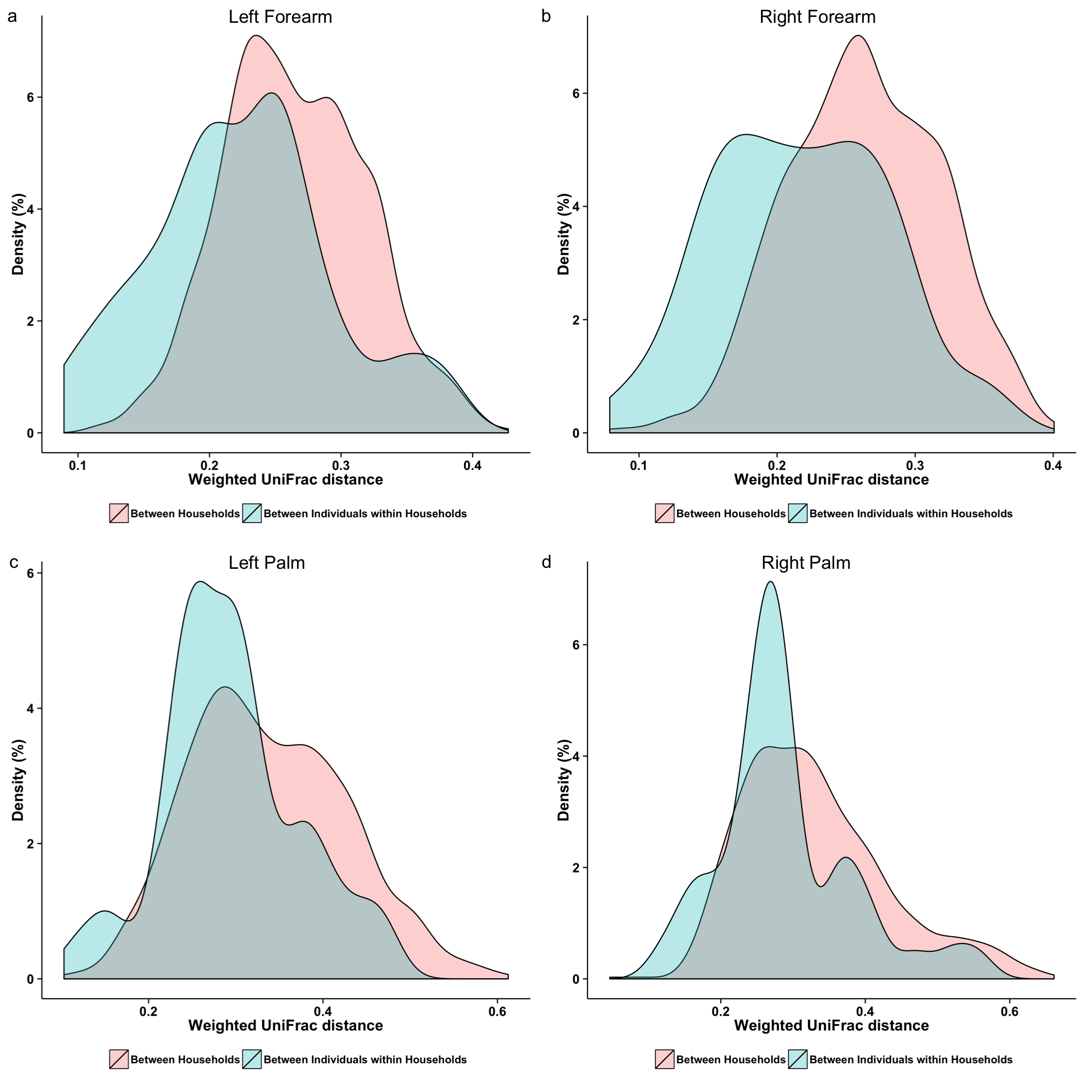


**Supplementary Fig. 3: Community structure dissimilarity between individuals by forearm and palm sites separated by symmetry.** Density plots of community structure dissimilarities represented by weighted UniFrac phylogenetic distances between individuals for (a) left and (b) right forearm, as well as (c) left and (d) right palm sites. Areas under curves are colour shaded depending on the relationships of samples under comparison (i.e. samples between individuals within a household, or samples between households).


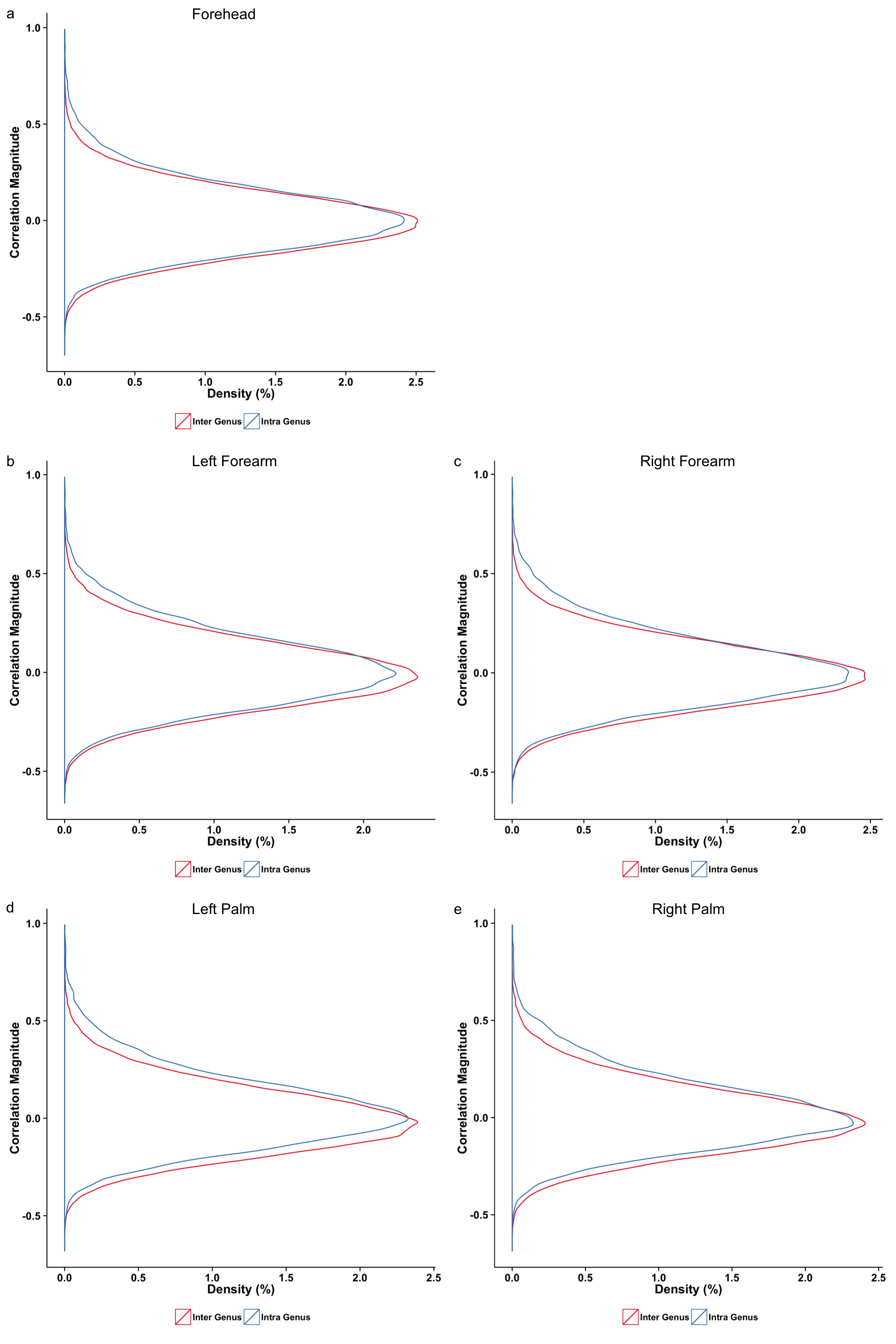


**Supplementary Fig. 4: Inter- and intra-genus co-abundance and co-exclusion magnitude comparisons.** Density plots of SparCC co-abundance and co-exclusion magnitudes between OTUs of the different (red) and same (blue) genera. Magnitude > 0 and < 0 represents co-abundance and co-exclusion relationships respectively. Plots generated for (a) forehead, (b) left and (c) right forearm, and (d) left and (e) right palm sites based on significant correlations (*p* ≤ 0.05 following bootstrapping).


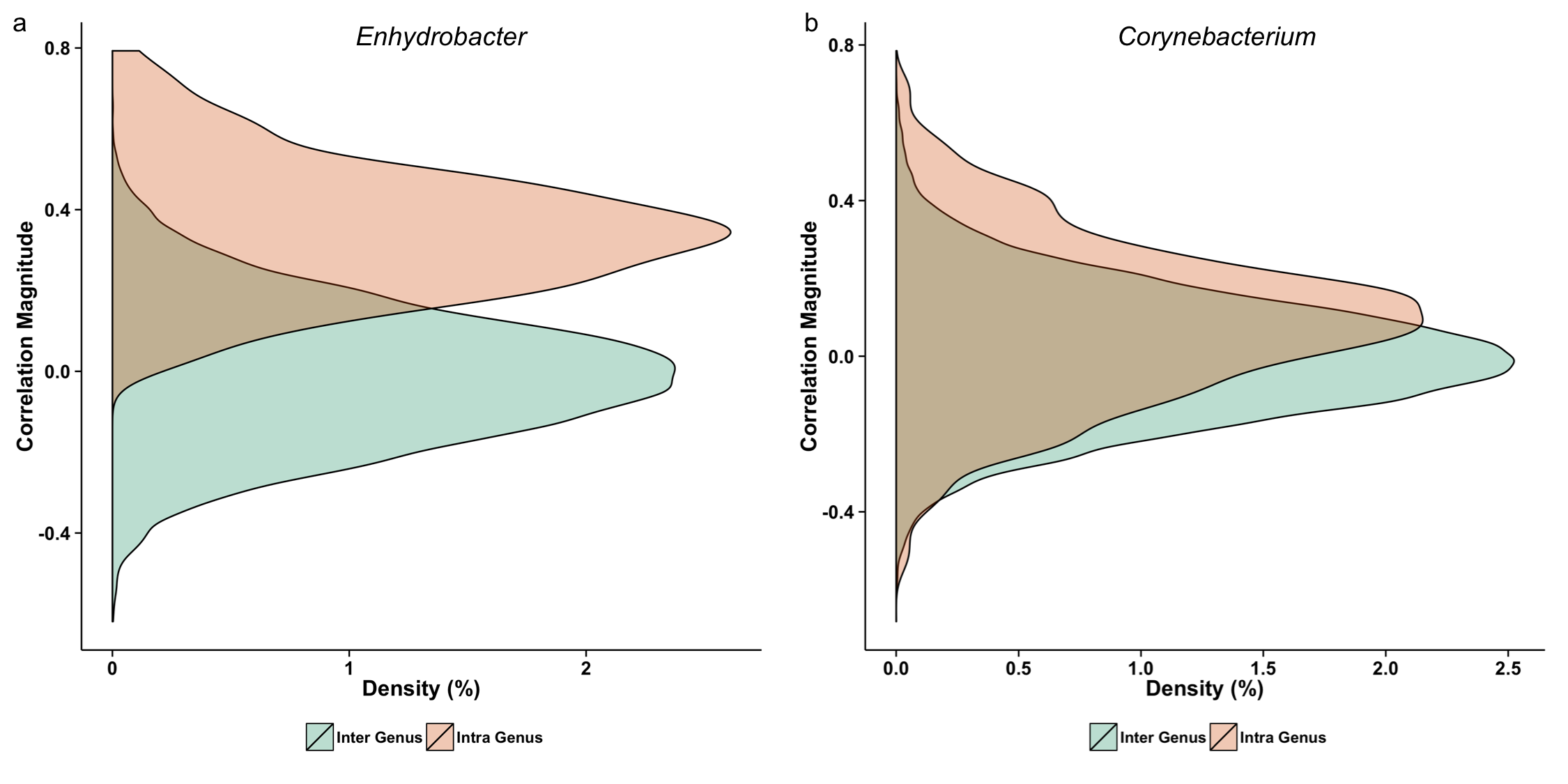


**Supplementary Fig. 5: Inter- and intra-genus significant relationships between (a) *Enhydrobacter* and (b) *Corynebacterium* on the forehead.** Density plots of SparCC significant *Enhydrobacter* and *Corynebacterium* co-abundance and co-exclusion magnitudes between OTUs of the different (green) and same (beige) genera. Magnitude > 0 represents co-abundance relationships, where < 0 represents co-exclusion relationships. Plots generated based on significant correlations (two-sided pseudo *p* ≤ 0.05 based on bootstrapping of 100 repetitions).

**Supplementary Tables**

|  | **Fold changes in % abundance^a^** | | | | |  |
| --- | --- | --- | --- | --- | --- | --- |
| **Genus** | **Forehead** | **Left Forearm** | **Right Forearm** | **Left Palm** | **Right Palm** | |
| ***Propionibacterium*** | 34.1 | 44.9 | 32.8 | 111.9 | 67.9 | |
| ***Staphylococcus*** | 81.4 | 109.2 | 36.4 | 229.7 | 227.8 | |
| ***Acinetobacter*** | 67.5 | 123.9 | 104.1 | 230.6 | 148.3 | |
| ***Streptococcus*** | 25.2 | 44.4 | 35.0 | 135.5 | 156.6 | |
| ***Enhydrobacter*** | 71.6 | 75.9 | 76.1 | 1169.2 | 715.4 | |
| ***Corynebacterium*** | 27.3 | 74.4 | 47.1 | 205.5 | 199.0 | |

^a^ Fold change determined by the sample with the highest abundance for the particular genus/site, divided by the sample with the lowest abundance for the particular genus/site

**Supplementary Table 1: Fold changes in relative abundances of the top six bacterial genera between individuals across sites**

| **Comparison^a^** | **Observed^b^** | **FPD^c^** | **Chao1** |
| --- | --- | --- | --- |
| Location | *P* = 1 x 10^-7^ | *P* = 8 x 10^-8^ | *P* = 3 x 10^-5^ |
| Anatomy (Symmetry combined) | *P* = 0.0001 | *P* = 0.0002 | *P* = 9 x 10^-6^ |
| Skin Site (Symmetry separated) | *P* = 0.001 | *P* = 0.002 | *P* = 9 x 10^-5^ |
| Gender | *P* = 0.001 | *P* = 0.0006 | *P* = 0.03 |
| Ventilation | *P* = 0.003 | *P* = 0.0009 | *P* = 0.05 |
| Age Group | NS | NS | *P* = 0.05 |

^a^ Comparison between gender and ventilation modes were computed using Mann-Whitney test, and Kruskal-Wallis was computed for the other comparisons

^b^ NS: statistically non-significant (P > 0.05)

^c^ FPD: Faith's phylogenetic distance

**Supplementary Table 2: Statistical significances of pairwise and multiple α-diversity comparisons between variables**

**Supplementary Information**

**Supplementary Table 1:** Fold changes in relative abundances of the top six bacterial genera between individuals across sites

**Supplementary Table 2.** Statistical significances of pairwise and multiple α-diversity comparisons between variables

**Supplementary Fig. 1:** **Rarefaction plots on observed number of OTUs.** Number of OTUs tabulated based on ≥ 97% cut-off to Greengenes database is plotted against number of sequence reads (sequencing depths) for each sample. Samples are coloured by (a) gender, (b) age group, and (c) sampling site. For all plots, the maximum depth shown in figure is 37,460 reads per sample, corresponding to the median read depth across all samples before rarefaction. Ten increments between depths of 10 and 37,460 reads inclusive were selected, and for each increment, the average richness of ten measurements was used to construct each curve. α-diversity comparisons performed in main text were based on a normalized read depth of 13,420 reads per sample (purple vertical line) to account for read depth differences between samples.

**Supplementary Fig. 2: Community structure dissimilarity between individuals by symmetry-combined skin sites.** Density plots of community structure dissimilarities represented by weighted UniFrac phylogenetic distances between individuals for (a) forehead, (b) symmetry-combined forearm, and (c) symmetry-combined palm sites. Areas under curves are colour shaded depending on the relationships of samples under comparison (i.e. samples from same individuals, samples between individuals within a household, or samples between households).

**Supplementary Fig. 3: Community structure dissimilarity between individuals by forearm and palm sites separated by symmetry.** Density plots of community structure dissimilarities represented by weighted UniFrac phylogenetic distances between individuals for (a) left and (b) right forearm, as well as (c) left and (d) right palm sites. Areas under curves are colour shaded depending on the relationships of samples under comparison (i.e. samples between individuals within a household, or samples between households).

**Supplementary Fig. 4: Inter- and intra-genus co-abundance and co-exclusion magnitude comparisons.** Density plots of SparCC co-abundance and co-exclusion magnitudes between OTUs of the different (red) and same (blue) genera. Magnitude > 0 and < 0 represents co-abundance and co-exclusion relationships respectively. Plots generated for (a) forehead, (b) left and (c) right forearm, and (d) left and (e) right palm sites based on significant correlations (*p* ≤ 0.05 following bootstrapping).

**Supplementary Fig. 5: Inter- and intra-genus significant relationships between (a) *Enhydrobacter* and (b) *Corynebacterium* on the forehead.** Density plots of SparCC significant *Enhydrobacter* and *Corynebacterium* co-abundance and co-exclusion magnitudes between OTUs of the different (green) and same (beige) genera. Magnitude > 0 represents co-abundance relationships, where < 0 represents co-exclusion relationships. Plots generated based on significant correlations (two-sided pseudo *p* ≤ 0.05 based on bootstrapping of 100 repetitions).

**Supplementary Data 1:** Sample metadata including individual and household characteristics

**Supplementary Data 2:** Relative abundances of major genera and Mann-Whitney and Kruskal-Wallis comparisons between gender, household ventilation, and age group

**Supplementary Data 3:** Core, distributed, and skin-site unique genera and OTUs with taxonomic classification

**Supplementary Data 4:** Relative abundances of Staphylococcal and Streptococcal species as classified by PATRIC pathogens database

**Supplementary Data 5:** α-diversity and Good's coverage across samples based on rarefaction of 13,420 reads per sample

**Supplementary Data 6:** Significant SparCC co-abundance or co-exclusion relationships between OTUs by body sites
